# Monitoring of Internet Forums to Evaluate Reactions to the Introduction of Reformulated OxyContin to Deter Abuse

**DOI:** 10.2196/jmir.3397

**Published:** 2014-05-02

**Authors:** Emily C McNaughton, Paul M Coplan, Ryan A Black, Sarah E Weber, Howard D Chilcoat, Stephen F Butler

**Affiliations:** ^1^Inflexxion, Inc.Newton, MAUnited States; ^2^Purdue Pharma L.P.Stamford, CTUnited States; ^3^Perelman School of MedicineUniversity of PennsylvaniaPhiladelphia, PAUnited States; ^4^Center for Psychological StudiesNova Southeastern UniversityFort Lauderdale, FLUnited States; ^5^Johns Hopkins Bloomberg School of Public HealthBaltimore, MDUnited States

**Keywords:** Internet, opioid analgesic, drug abuse, prescription drug, OxyContin, epidemiology, surveillance, social media, qualitative research

## Abstract

**Background:**

Reformulating opioid analgesics to deter abuse is one approach toward improving their benefit-risk balance. To assess sentiment and attempts to defeat these products among difficult-to-reach populations of prescription drug abusers, evaluation of posts on Internet forums regarding reformulated products may be useful. A reformulated version of OxyContin (extended-release oxycodone) with physicochemical properties to deter abuse presented an opportunity to evaluate posts about the reformulation in online discussions.

**Objective:**

The objective of this study was to use messages on Internet forums to evaluate reactions to the introduction of reformulated OxyContin and to identify methods aimed to defeat the abuse-deterrent properties of the product.

**Methods:**

Posts collected from 7 forums between January 1, 2008 and September 30, 2013 were evaluated before and after the introduction of reformulated OxyContin on August 9, 2010. A quantitative evaluation of discussion levels across the study period and a qualitative coding of post content for OxyContin and 2 comparators for the 26 month period before and after OxyContin reformulation were conducted. Product endorsement was estimated for each product before and after reformulation as the ratio of endorsing-to-discouraging posts (ERo). Post-to-preintroduction period changes in ERos (ie, ratio of ERos) for each product were also calculated. Additionally, post content related to recipes for defeating reformulated OxyContin were evaluated from August 9, 2010 through September 2013.

**Results:**

Over the study period, 45,936 posts related to OxyContin, 18,685 to Vicodin (hydrocodone), and 23,863 to Dilaudid (hydromorphone) were identified. The proportion of OxyContin-related posts fluctuated between 6.35 and 8.25 posts per 1000 posts before the reformulation, increased to 10.76 in Q3 2010 when reformulated OxyContin was introduced, and decreased from 9.14 in Q4 2010 to 3.46 in Q3 2013 in the period following the reformulation. The sentiment profile for OxyContin changed following reformulation; the post-to-preintroduction change in the ERo indicated reformulated OxyContin was discouraged significantly more than the original formulation (ratio of ERos=0.43, *P*<.001). A total of 37 recipes for circumventing the abuse-deterrent characteristics of reformulated OxyContin were observed; 32 were deemed feasible (ie, able to abuse). The frequency of posts reporting abuse of reformulated OxyContin via these recipes was low and decreased over time. Among the 5677 posts mentioning reformulated OxyContin, 825 posts discussed recipes and 498 reported abuse of reformulated OxyContin by such recipes (41 reported injecting and 128 reported snorting).

**Conclusions:**

After introduction of physicochemical properties to deter abuse, changes in discussion of OxyContin on forums occurred reflected by a reduction in discussion levels and endorsing content. Despite discussion of recipes, there is a relatively small proportion of reported abuse of reformulated OxyContin via recipes, particularly by injecting or snorting routes. Analysis of Internet discussion is a valuable tool for monitoring the impact of abuse-deterrent formulations.

## Introduction

Prescription opioid analgesics are an important component of pain management. Misuse and abuse of these medications, however, have created a serious and growing public health problem [1]. The balance between providing access to and prescribing these medications for patients with chronic pain while minimizing their diversion and abuse remains a significant challenge for all stakeholders, including prescribers, pharmaceutical manufacturers, and the Food and Drug Administration [2,3]. One important step toward the goal of creating safer opioid analgesics has been the development of opioid formulations designed to deter abuse [4-6]. These formulations are commonly referred to as abuse-deterrent formulations (ADFs) [7] or tamper-resistant formulations (TRFs). The science of deterring abuse via these formulations is new, and both the formulation technologies and the analytical, clinical, epidemiological, and statistical methodology for evaluating those technologies are rapidly evolving.

Most abuse-deterrent technologies developed to date are designed to make product manipulation more difficult or to make abuse of the manipulated product less attractive or rewarding. Although in vitro and clinical studies indicate the efficacy of these technologies, postmarketing data are needed to evaluate their effectiveness. One of the early formulations intended to reduce abuse was a reformulated version of extended-release oxycodone (reformulated OxyContin, Purdue Pharma, Stamford, CT, USA), which was introduced to the market in August 2010. This product has physicochemical resistance to crushing and dissolution intended to present obstacles to abuse by nonoral routes of administration (ROA) (eg, injecting, snorting). The launch of reformulated OxyContin provided a nationwide experiment to evaluate the impact of a product intended to reduce tampering in the real world [8,9]. To date, evidence from individuals evaluated for treatment triage suggests that reformulated OxyContin results in lower rates of abuse through nonoral abuse and abuse via any ROA [8] compared to historical rates for the original formulation of OxyContin. These findings, as well as others [10,11] that suggest reformulated OxyContin inhibits manipulation and abuse, are based on reports by abusers to some authority (eg, researcher, treatment provider, poison control center). The question arises as to the reaction to reformulated OxyContin of individuals who abuse prescription opioids and are not reporting abuse to researchers or other authorities. It is of further interest to monitor and describe the extent to which individuals are engaging in efforts to defeat the tamper-resistant properties of reformulated OxyContin and whether such efforts were deemed feasible.

Introduction of reformulated OxyContin presents an opportunity to determine the utility of monitoring Internet data to evaluate reactions to this formulation among a difficult-to-reach population of prescription drug abusers who are not generally in contact with some authority [12]. Because these Internet data reflect uninhibited peer-to-peer communications, they may be a useful source for monitoring and tracking efforts to defeat the abuse-deterrent properties of the product for illicit use. It is generally believed that these efforts will take the form of “recipes” that will be disseminated via the Internet [13-15]. Furthermore, it is anticipated that the feasibility and utility of a recipe will be evaluated by abusers online and that practical tampering methods will be disseminated and perpetuated through postings on websites dedicated to recreational abuse of drugs [15]. Based on this scenario, public health stakeholders are increasingly concerned about monitoring discussions around extraction techniques that emerge on the Internet and tracking the dissemination of these methods [2].

Although public Internet forums can be monitored unobtrusively and might reveal ways in which prescription drugs are being misused [16], there has been little published to date on how to collect, analyze, and understand the messages within the large volume of posts available from online recreational drug abuse communities. Early studies [17,18] that examined the feasibility of systematic Internet surveillance of discussion of prescription opioid products indicated that Internet posts can be reliably coded for sentiment (eg, endorsing vs discouraging abuse) and that both the amount of discussion and sentiment differentiated products [18]. In subsequent work, McNaughton et al [12] developed a metric, referred to as the endorsement ratio (ERo), to evaluate and quantify the overall sentiment expressed by a large number of opioid abusers who post online about prescription opioid products.

In the present work, we sought to understand how drug abusers reacted to the introduction of an intended tamper-resistant prescription opioid product to the market. We examined data from abusers who participated in Internet message boards to evaluate discussion of OxyContin before and after introduction of the reformulation. Specifically, we investigated these questions: (1) did the level of Internet discussion related to OxyContin change quantitatively over time following introduction of the reformulated version of the product, (2) within the OxyContin-specific discussion that did occur, was there a shift in the sentiment expressed by abusers who posted on these websites following the introduction of reformulated OxyContin, and (3) given concerns about efforts to generate and disseminate tampering methods intended to defeat the properties of reformulated OxyContin for use by unintended ROAs, could Internet discussion of such recipes be defined, identified, and monitored?

## Methods

### Study Overview

The study aimed to evaluate the potential effect the introduction of the reformulation of OxyContin had on discussion within Internet-based recreational drug abuse message boards. Over the pre-post reformulated OxyContin timeframe, we conducted (1) a quantitative evaluation of message board discussion for OxyContin and comparators to capture the relative levels of discussion and any changes during the pre-post time period, (2) a qualitative coding of Internet post content and estimation of endorsement for OxyContin and comparators to determine any changes in the sentiment in favor of each medication for abuse purposes from pre to post OxyContin reformulation, and (3) in the period following the introduction of OxyContin, an evaluation of Internet post content related to tampering methods for defeating the abuse-deterrent properties of reformulated OxyContin. All research activities conducted for this study were exempt from Institutional Review Board review as determined by the New England Institutional Review Board.

For the quantitative evaluation of discussion levels and content analysis/estimation of endorsement, Vicodin (hydrocodone) and Dilaudid (hydromorphone) were selected as comparators. These comparators represented a widely available and highly abused prescription opioid (Vicodin) and a high-potency opioid analgesic that is highly desirable for abuse (Dilaudid) [19]. In order to make appropriate comparison to the target product (OxyContin), qualitative coding and analysis was restricted to discussion of the proprietary products Vicodin and Dilaudid only and did not include generic references to hydrocodone, hydromorphone, and other proprietary products within the opioid compounds (eg, Lortab for hydrocodone and Exalgo for hydromorphone).

### Data Source

The study sample consisted of Internet posts (ie, messages) copied from 7 publically accessible message boards that represent a population of drug abusers and their online communications regarding both illicit and prescription drugs. The websites were chosen based upon predefined criteria as described in McNaughton et al [12]. All posts written between January 1, 2008 and September 30, 2013 (N=6,891,514) were archived in a database for further sampling and analysis. No personal identifiable information related to the author was retained.

### Quantitative Evaluation of Message Board Discussion

From the database of saved Internet posts, all messages related to OxyContin (both original and reformulated versions of the product), Vicodin, and Dilaudid written between January 1, 2008 and September 30, 2013 (ie, Q1 2008 through Q3 2013) were identified through the use of standardized queries. These queries contained text-matching criteria that included common misspellings, slang, and wildcard characters as well as exclusion criteria to capture as many relevant posts as possible while minimizing the number of false positives (ie, posts returned by the query that are not actually related to the target product) selected. It should be noted, however, that false positives could not be completely eliminated from the text-matching query results without manual review, which was not conducted for this analysis because of the magnitude of posts involved. The rate of discussion related to each product was then calculated as the number of product-specific posts identified per 1000 posts saved within the database per quarter.

### Formal Content Analysis and Estimation of Endorsement

A formal content analysis was conducted on random samples of Internet posts related to OxyContin, Vicodin, and Dilaudid during the 26-month period before (preintroduction period=June 1, 2008 through July 30, 2010) and the 26-month period after the introduction of reformulated OxyContin (postintroduction period=August 1, 2010 through September 30, 2012) and identified through the use of the standardized queries. For this analysis, posts retained for coding in the preintroduction period pertained to the original formulation of OxyContin, whereas posts sampled and retained in the postintroduction period related specifically to reformulated OxyContin. Because the design involved comparison of discussion of original OxyContin in the preintroduction period and reformulated OxyContin in the postintroduction period, discussion of original OxyContin in the postintroduction period was not examined for this study. Using systematic query searches, product-specific Internet posts were randomly sampled from the archive. All coding was conducted as part of a larger dynamic postmarketing surveillance program, involving rolling sampling and content analysis of posts (ie, multiple waves of sampling throughout the study period). Power analyses to determine the sample size needed to detect changes were calculated periodically throughout surveillance and changed over time resulting in somewhat different sample sizes in the preintroduction and postintroduction periods for this evaluation.

**Figure 1:**
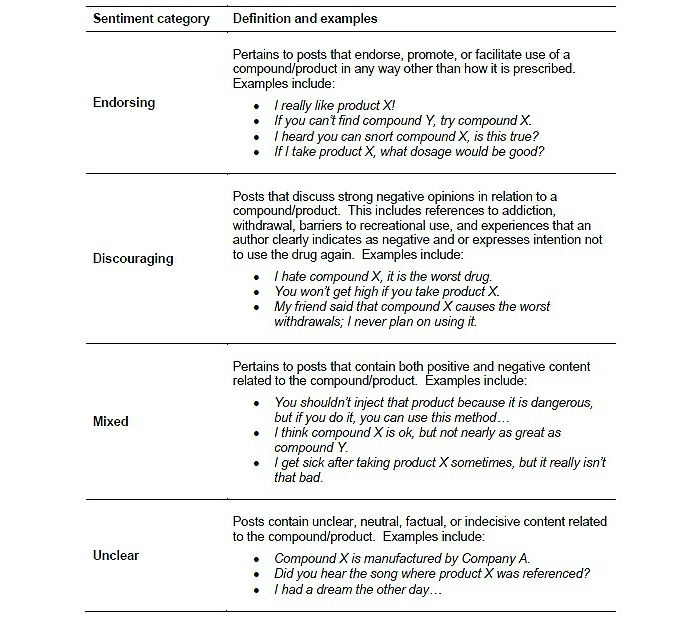
Abuse-related sentiment categories in formal content analysis.

The coding procedure and assessment of intercoder agreement used in this study is described in detail in McNaughton et al [12]. Briefly, posts were reviewed by trained coders and categorized as either abuse-related or non-abuse-related, and false positives were removed and replaced. A false positive is a query-selected post that upon manual review did not pertain to the specified prescription opioid product. Within the sample of abuse-related posts, product-specific content was further coded as endorsing, discouraging, mixed, or unclear (ie, the sentiment was assigned) (Figure 1). When there was disagreement between coders, the post content was discussed and reviewed by an independent lead coder for a final rating and to achieve a final set of codes for analysis. To assess reliability of the coding, 20% of all posts were coded by 2 coders who were blinded to which posts were coded by both coders and which were coded independently. Interrater agreement (kappa) was then calculated on the 20% overlapping sample to determine if an acceptable level of coder reliability was achieved [20].

A mixed effects multinomial logistic regression was employed to model the probability of observing each of the 4 types of abuse-related Internet posts (endorsing, discouraging, mixed, and unclear) per product. The fixed effects included a product indicator (1=product A, 2=product B, etc), time indicator (1=preintroduction period, 2=postintroduction period) and product×time interaction. An author random effect was incorporated in the model to account for correlation among messages posted by the same author. The GLIMMIX procedure in SAS 9.3 (SAS Institute, Inc, Cary, NC, USA) was used to fit the model, producing the following statistics of interest:

1. Probability of observing each type of abuse-related post (endorsing, discouraging, mixed, and unclear) per product in the period before and after the introduction of reformulated OxyContin.
2. Endorsement ratio (ERo) for each product in the period before and after the reformulation of OxyContin. The ERo provides a relative estimate of the extent to which a product was being endorsed during each time period by calculating a ratio of probabilities (eg, probability of endorsing product A in the postintroduction period divided by probability of discouraging product A in the postintroduction period), commonly referred to as a relative risk [12].
3. Post-to-preintroduction change in the ERo was estimated by calculating the ratio of ERos (eg, ERo of product A in the postintroduction period divided by ERo of product A in the preintroduction period), commonly referred to as a relative risk ratio.
4. Within-author correlation as estimated by intraclass correlation coefficients derived from the variance components [21].

### Evaluation of Recipes

Of particular interest with respect to any purported ADF product is whether tampering methods, or recipes, are developed that allow individuals to readily defeat the abuse-deterrent properties of a new formulation. To evaluate this possibility, a review of recipe-related content was performed on Internet posts pertaining to reformulated OxyContin during the approximately 3-year period following the product’s launch (August 9, 2010 through September 30, 2013). For this evaluation, a recipe was defined as a process (physical, chemical, or potentiation) that enabled use of the product in a way other than intended (ie, swallowing a tablet whole) because ADFs are not formulated to prevent abuse by swallowing multiple tablets whole at one time [4,5]. While variation existed with respect to (1) the format in which a recipe was communicated (eg, step-by-step instruction guide vs narrative experience report), (2) the words used to describe a recipe, and (3) the devices used by an individual for a particular recipe; recipes were classified into profiles that represented the fundamental or basic steps used when manipulating a product. For example, 2 posts, one that references “crushing a tablet with a knife before putting in water” and a second that notes “(1) pound product with a hammer, (2) add water” would be classified as the same recipe profile (ie, crush and dissolve) despite differences in the format, words, and devices communicated.

All posts that referenced OxyContin during the approximately 3-year period were reviewed by a trained coder for recipe content related to the reformulated version of OxyContin. For each post that mentioned a recipe related to reformulated OxyContin, the coder assigned 3 codes: (1) the recipe profile, (2) the ROA mentioned in relation to the recipe profile, and (3) whether the author described the recipe as “feasible.” Feasibility was defined as being able to manipulate reformulated OxyContin for abuse via an unintended ROA (ie, use of the product other than swallowing the tablet whole). Utilizing the coded information, the total number of recipe-related posts, recipe profiles, and the frequency in which recipe profiles were first observed are presented. In addition, the ROAs mentioned in relation to feasible recipes are provided.

## Results

### Quantitative Evaluation of Message Board Discussion

**Figure 2:**
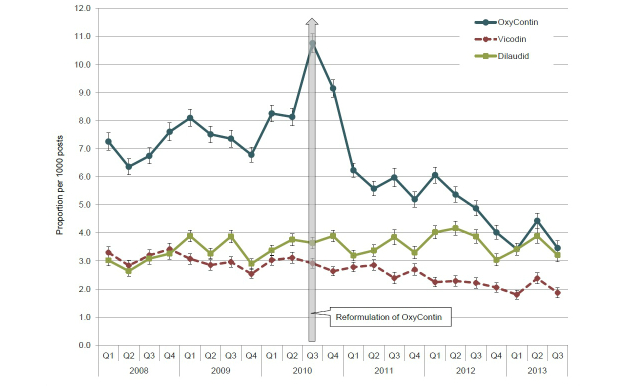
Proportion of OxyContin-, Vicodin-, and Dilaudid-related posts from Q1 2008 to Q3 2013.

Between January 1, 2008 and September 30, 2013 (ie, Q1 2008 through Q3 2013), 45,936 posts related to OxyContin (original formulation in the preintroduction period and both original and reformulated versions of the product in the postintroduction period) were identified in the database of 6,891,514 saved posts. Because the brand name of OxyContin did not change following the introduction of the reformulation, it was not possible to disambiguate references to original versus reformulated OxyContin in the postintroduction period without review of each post, which was not conducted for this analysis. In addition, 18,685 posts related to Vicodin, and 23,863 posts related to Dilaudid were identified. When evaluated by quarter, the proportion of OxyContin-related posts fluctuated between 6.35 and 8.25 posts per 1000 posts during the period before the release of reformulated OxyContin (Q1 2008 through Q2 2010) before increasing to an observed 10.76 posts per 1000 posts in Q3 2010 with the launch of reformulated OxyContin on August 9, 2010 (Figure 2). Following the release of reformulated OxyContin, the proportion of OxyContin posts remained elevated at 9.15 posts per 1000 posts in Q4 2010 before decreasing in Q1 2011 (6.23 posts per 1000). From Q1 2011 through Q3 2013, the proportion of OxyContin-related posts decreased over time, from 6.23 posts per 1000 posts in Q1 2011 to 3.46 posts per 1000 posts in Q3 2013 and remained consistently lower than the quarterly proportions observed before the release of the reformulation (Q2 2008 through Q2 2010). Changes in the proportion of OxyContin-related posts before and following release of the reformulated version of the product, however, contrast with the comparatively consistent pattern of discussion observed for both Vicodin (range 1.87-3.30 posts per 1000 posts) and Dilaudid (range 2.64-4.16 posts per 1000 posts) during the same time period (Figure 2).

### Formal Content Analysis and Estimation of Endorsement

#### Coding Results

Of the 16,588 posts sampled, 5365 (32.40%) were identified as false positives and removed from the final sample (Table 1). The high false positive rate for the entire sample was primarily attributable to the number of false positive posts associated with reformulated OxyContin in the postintroduction period. Using the rolling sampling procedures, a total of 11,223 posts were retained for analysis: 3741 posts for each product. For the 20% overlapping sample (ie, posts coded by both reviewers), kappa was calculated on (1) whether a post was abuse-related and, if abuse-related, (2) whether the content was endorsing, discouraging, mixed, or unclear (ie, the sentiment). Kappa statistics were calculated by product and period as well as across all compounds. All kappas were satisfactory (Table 1) [20].

**Table 1:** Content analysis: number of posts sampled and retained, false positive rate, and interrater agreement kappa statistics.

Product and period^a^		Total posts sampled, n	False positives, n (%)	Final sample, n	Kappa	
					Abuse-related	Sentiment
**OxyContin** ^b^						
	Pre	2256	287 (12.72)	1969	.82	.68
	Post	5750	3978 (69.18)^c^	1772	.87	.77
**Vicodin**						
	Pre	2226	257 (11.55)	1969	.78	.64
	Post	2053	281 (13.69)	1772	.78	.63
**Dilaudid**						
	Pre	2233	264 (11.82)	1969	.83	.68
	Post	2070	298 (14.40)	1772	.75	.65
Total		16,558	5365 (32.40)	11,223	.87	.72

^a^Preintroduction period: the period before the reformulation of OxyContin (June 1, 2008 through July 31, 2010); postintroduction period: the period following the reformulation of OxyContin (August 1, 2010 through September 30, 2012).

^b^Preintroduction period represents content related to the original formulation of OxyContin. Postintroduction period represents content related to the reformulated version of OxyContin.

^c^The high false positive rate observed for reformulated OxyContin during the postintroduction period was related to the slang term “OP” (ie, the indicia on the reformulated tablet) which is also an acronym commonly used on message boards to refer to the “original poster” or the first author to write a post in a thread. Furthermore, the standardized queries sometimes yielded posts in which the discussion could not clearly be identified as pertaining to the reformulated version of OxyContin specifically, which resulted in a high degree of false positives.

#### Estimation of Endorsement

The probability of observing endorsing, discouraging, mixed, or unclear abuse-related sentiments for OxyContin, Vicodin, and Dilaudid in the periods before (preintroduction period) and following (postintroduction period) the introduction of reformulated OxyContin is presented in Table 2. Overall, the probability of observing content related to each sentiment category varied by product and period. For OxyContin specifically, the probability of observing posts with endorsing sentiment was greater for the original formulation (preintroduction period: probability=0.43) than for the reformulated version (postintroduction period: probability=0.22). Conversely, the probability of observing a discouraging post was lower for the original formulation in the preintroduction period (probability=0.22) than for the reformulated version in the postintroduction period (probability=0.27). When evaluated as an ERo [12] as a means of estimating the extent to which the product was endorsed, in the period before the release of reformulated OxyContin, the probability of observing posts that endorsed the use of the original formulation of OxyContin was approximately 1.91 times greater than the probability of discouraging the product (Table 3). In the postintroduction period, however, reformulated OxyContin was 1.23 times more likely to be discouraged than endorsed (ERo=0.81). Taken together, the change in the ERo estimates before and after the introduction of reformulated OxyContin indicate that the ERo for the original formulation of OxyContin in the preintroduction period was 2.33 times greater than the ERo estimate for the reformulated version of OxyContin in the postintroduction period (ratio of ERos=0.43, *P*<.001) (Table 3).

**Table 2:** Abuse-related sentiment category probabilities.

Product and period^a^		Endorsing		Discouraging		Mixed		Unclear	
		Prob	95% CI	Prob	95% CI	Prob	95% CI	Prob	95% CI
**OxyContin** ^b^									
	Pre	0.43	0.40-0.45	0.22	0.20-0.25	0.18	0.16-0.20	0.16	0.14-0.18
	Post	0.22	0.20-0.24	0.27	0.24-0.30	0.23	0.21-0.25	0.28	0.25-0.31
**Vicodin**									
	Pre	0.36	0.33-0.38	0.29	0.26-0.31	0.22	0.20-0.24	0.13	0.12-0.15
	Post	0.35	0.32-0.37	0.17	0.15-0.19	0.28	0.26-0.30	0.20	0.18-0.22
**Dilaudid**									
	Pre	0.46	0.43-0.49	0.19	0.17-0.22	0.25	0.23-0.28	0.09	0.08-0.11
	Post	0.47	0.44-0.49	0.09	0.08-0.11	0.31	0.28-0.33	0.13	0.11-0.15

^a^Preintroduction period: the period before the reformulation of OxyContin (June 1, 2008 through July 31, 2010); postintroduction period: the period following the reformulation of OxyContin (August 1, 2010 through September 30, 2012).

^b^Preintroduction period represents content related to the original formulation of OxyContin. Postintroduction period represents content related to the reformulated version of OxyContin.

**Table 3:** Endorsement ratios (ERo) and post-to-preintroduction period ratios of Eros.

Product and period^a^		ERo^b^	95% CI	Ratio of ERos^c^	95% CI	*P*
**OxyContin** ^d^				0.43	0.35-0.52	<.001
	Pre	1.91	1.66-2.20			
	Post	0.81	0.69-0.95			
**Vicodin**				1.66	1.36-2.04	<.001
	Pre	1.24	1.08-1.42			
	Post	2.06	1.76-2.43			
**Dilaudid**				2.11	1.68-2.63	<.001
	Pre	2.38	2.05-2.77			
	Post	5.01	4.15-6.05			

^a^Preintroduction period: the period before the reformulation of OxyContin (June 1, 2008 through July 31, 2010); postintroduction period: the period following the reformulation of OxyContin (August 1, 2010 through September 30, 2012).

^b^The ERo is a ratio of probabilities (eg, probability of endorsing product A in the postintroduction period divided by probability of discouraging product A in the postintroduction period), which is commonly referred to as a relative risk.

^c^The post-to-preintroduction ratio of ERos is an estimate of the change the ERo before and after the introduction of reformulated OxyContin (eg, ERo of product A in the postintroduction period divided by ERo of product A preintroduction period), which is commonly referred to as a relative risk ratio.

^d^Preintroduction period represents content related to the original formulation of OxyContin. Postintroduction period represents content related to the reformulated version of OxyContin.

Changes in the sentiment profiles of Vicodin and Dilaudid were also observed before and after the introduction of the reformulated version of OxyContin. In relation to Vicodin, the ERo was 1.66 times greater in the postintroduction period than in the preintroduction period (*P*<.001) indicating that the ratio of encouraging-to-discouraging discussion for Vicodin in the period following the introduction of reformulated OxyContin was significantly greater than in the period before the reformulation (Table 3). Likewise, the ERo for Dilaudid was 2.11 times greater (*P*<.001) in the postintroduction period than in the preintroduction period. These changes in the ERo estimate for Vicodin and Dilaudid, however, appear to be because of a reduction in posts coded as discouraging rather than an increase in encouraging posts. In relation to the post-to-preintroduction period ratio of the ERos for Vicodin and Dilaudid compared to OxyContin, however, the magnitude of the change for Vicodin and Dilaudid was 3.91 times greater (*P*<.001) and 4.95 times greater (*P*<.001) than OxyContin, respectively. The post-to-preintroduction period ratio of the ERo estimates for Vicodin compared to Dilaudid was not statistically different (*P*=.12). These results suggest that the endorsing and discouraging sentiment profile for OxyContin before and after the introduction of the reformulation changed significantly more than the endorsing and discouraging sentiment profile of Vicodin and Dilaudid.

### Evaluation of Recipes

**Figure 3:**
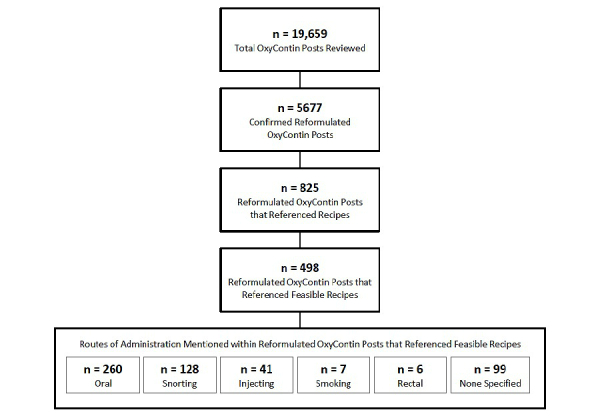
Evaluation of recipes: coding results. Feasibility was defined as being able to manipulate reformulated OxyContin for abuse via an unintended route of administration (ie, use of product other than swallowing the tablet whole).

**Figure 4:**
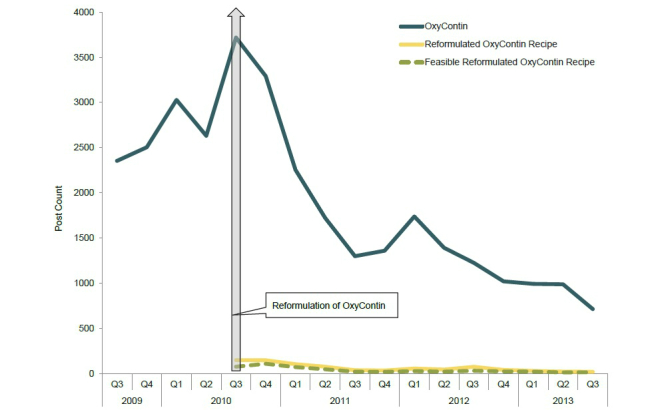
Frequencies of OxyContin-, reformulated OxyContin recipe-, and feasible reformulated OxyContin recipe-related posts from Q3 2009 to Q3 2013. For the reformulated OxyContin recipe and feasible OxyContin recipe categories, Q3 2010 includes data from August 9, 2010 to September 30, 2010.

During the approximate 3-year period following the launch of reformulated OxyContin (August 9, 2010 through September 30, 2013), 19,659 posts related to OxyContin (both original and reformulated versions of the product) were identified and reviewed by trained coders (Figure 3). Of these, 5677 posts were identified as referring specifically to the reformulated version of OxyContin. Within this reformulated OxyContin-specific discussion, recipes related to reformulated OxyContin were mentioned 1052 times within 825 posts (14.5% of reformulated OxyContin-related discussion) and evidence of feasible manipulation of reformulated OxyContin (ie, use of the product other than swallowing the tablet whole) was observed 576 times within 498 posts (8.8% of reformulated OxyContin-related discussion) across the approximately 3-year period. As Figure 4 illustrates, the frequency of OxyContin-related posts peaked with the introduction of reformulated OxyContin and then declined steadily. Figure 4 also shows a general decrease over the approximately 3-year period in the number of posts specifically referencing a reformulated OxyContin recipe as well as posts that specifically mentioned a feasible recipe. An exception to this general decrease was a slight increase in Q1 2012, which is likely related to discussion associated with the launch of a reformulated version of extended-release oxymorphone. Specifically, authors discussed their experience with reformulated OxyContin recipes and whether or not those methods could be used with the reformulated version of extended-release oxymorphone.

**Figure 5:**
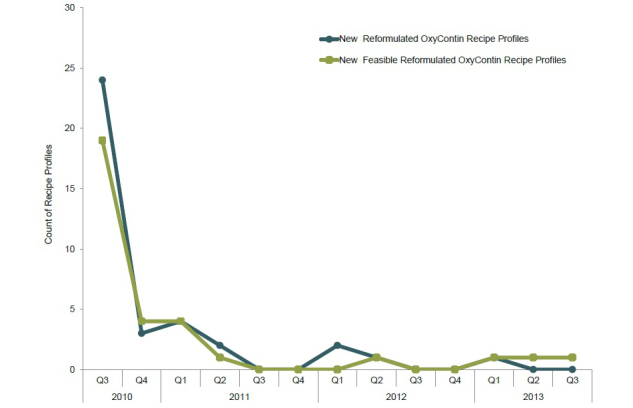
Frequency of new reformulated OxyContin recipe profiles and new feasible reformulated OxyContin recipe profiles.

In total, 37 unique recipe profiles were identified during the approximately 3-year period, 32 of which were denoted as feasible at least once (Table 4). Within the reformulated OxyContin recipe-related posts, most referenced 12 of the 37 profiles, whereas the remaining 25 were mentioned fewer than 10 times each during the approximately 3-year period. The frequency with which new recipe profiles emerged decreased following the first quarter after the launch of reformulated OxyContin (from 24 in Q3 2010 [ie, August 9, 2010 to September 30, 2010] to 3 in Q4 2010), and few new recipe profiles were identified in subsequent quarters (Figure 5). Likewise, the number of new feasible reformulated OxyContin recipe profiles observed over time followed a similar pattern.

When considering the 498 reformulated OxyContin-related posts that referenced a feasible recipe profile, various ROAs were mentioned in relation to use of the manipulated product (Figure 3). Oral use of reformulated OxyContin following feasible use of a recipe (eg, drinking in solution, chewing, parachuting) was mentioned in 4.58% (260/5677) of all reformulated OxyContin-related discussion, followed by snorting in 2.25% (128/5677) of reformulated-related discussion, and injection in 0.72% (41/5677) of reformulated OxyContin-related discussion. Smoking or rectal administration of reformulated OxyContin following feasible manipulation were observed 7 and 6 times, respectively, during the approximately 3-year period following the introduction of the reformulated version of OxyContin. It should be noted that an author could reference more than 1 recipe profile as well as more than 1 ROA in relation to a recipe profile within the same post; therefore, the ROA categories within the 498 posts that mentioned feasible recipe profiles are not mutually exclusive. Furthermore, some authors did not indicate use of a specific ROA (99/5677, 1.74%) following feasible manipulation.

**Table 4:** Frequency of reformulated OxyContin recipe profiles.

Recipe profile	Posts that mentioned recipe profile, n	Posts that mentioned recipe profile was feasible, n
Crush/shave	277	152
Dissolve/soak	130	58
Chew	114	81
Crush/shave, heat, and freeze	89	72
Crush/shave and dissolve/soak	71	39
Crisp	50	35
Crush/shave and heat	40	26
Crush/shave, heat, and dissolve/soak	25	17
Crush/shave, add chemicals, and evaporate	24	13
Take with acidic foods or beverages	23	19
Heat	19	6
Take with alcohol	10	6
Dissolve/soak and heat	9	4
Heat and freeze	8	2
Crush/shave, heat, freeze, and dissolve/soak	7	7
Crush/shave and freeze (or vice versa)	7	5
Crush/shave, add chemicals, dissolve, and filter	5	4
Take with a fatty meal	5	4
Crush/shave, heat, cool, dissolve/soak, and filter	4	3
Crush/shave, heat, dissolve/soak, and evaporate	4	3
Dissolve/soak and filter	4	1
Add soda, heat, and take with acidic beverage	4	3
Crisp, dissolve/soak, and filter	4	1
Dissolve/soak and freeze	3	0
Crush/shave, heat, dissolve/soak in chemical	3	0
Crush/shave, dissolve/soak, and evaporate	2	0
Crush/shave, heat, dissolve/soak, and filter	2	2
Heat, cool, crush/shave, dissolve/soak, and heat	2	3
Freeze, crush/shave, heat, and crush/shave	2	3
Crisp, heat, and freeze	2	1
Crush/shave, add chemicals, evaporate, and heat	2	1
Crush/shave, dissolve/soak, and cool/freeze	1	0
Heat, crush/shave, heat, and freeze	1	1
Crush/shave, add chemicals, evaporate, and cool	1	1
Dissolve/soak, heat, and filter	1	0
Crush/shave, heat, freeze, crisp, and filter	1	1
Crush/shave, dissolve/soak, add chemical	1	1
Total	825^a^	498^a^

^a^An author could reference more than 1 recipe profile within the same post; therefore, the total number of recipe-related posts does not equal the sum of the counts across the 37 recipe profiles.

## Discussion

### Principal Findings

Novel methodologies for evaluating the impact of prescription opioid products with abuse-deterrent properties on abuse-related behaviors are needed. This study presents an Internet-focused approach to examine discussion on recreational drug abuse Internet forums regarding reformulated OxyContin, the first widely available reformulated opioid product on the market. Systematic monitoring and review of content from online message boards before and after the introduction of reformulated OxyContin allowed for evaluation of (1) discussion about OxyContin and 2 comparators, (2) relative endorsement of OxyContin and comparators, and (3) discussions around manipulation of reformulated OxyContin through recipes.

Overall, the findings presented here suggest that the introduction of reformulated OxyContin had an impact on discussion of OxyContin on message boards frequented by prescription drug abusers. Quantitative analysis indicated that the volume of discussion related to OxyContin increased in the quarters leading up to the launch of the reformulated version of the product and subsequently decreased to levels lower than those observed in the period before the reformulation. In contrast, the level of discussion associated with the selected comparators (Vicodin, a widely available and highly abused prescription opioid, and Dilaudid, a high-potency opioid analgesic that is highly desirable for abuse [19]) remained consistent across the pre-postintroduction period. Content analyses revealed that sentiment related to OxyContin on the message boards changed after the introduction of the reformulation as reflected by a significant decline in the ERo for OxyContin following the reformulation of the product. That is, the online consensus regarding the desirability of OxyContin for abuse appears to have shifted from a positive sentiment to a relatively and significantly more negative view. Individuals who participated on the message boards reviewed during the study period expressed preference for the original version of OxyContin over the reformulated product as evidenced by both the shift in the sentiment profile and the overall decrease in the level of discussion associated with OxyContin over time during the postintroduction period.

The analyses of sentiment built upon prior work [12] by applying the endorsement ratio methodology; that is, using the ERo to quantify change in sentiment expressed by recreational drug abusers about a product with tamper-resistant properties (reformulated OxyContin) compared with sentiment expressed for the parent product (original formulation OxyContin). Our observations are also consistent with findings from other studies [8-11], including one of a sentinel surveillance sample of individuals assessed for substance use problems in the first 20 months after the introduction of reformulated OxyContin that found that the reformulation impacted abuse patterns of OxyContin [8].

Prior to the launch of any reformulated product, like reformulated OxyContin, concern existed that the product would be greeted with numerous attempts by abusers to defeat the product’s tamper-resistant mechanism. A particular concern was that any truly successful recipe would be widely and rapidly disseminated online [15]. Results of the systematic examination presented here suggest that abusers who posted online responded to the introduction of the reformulation with discussion related to recipes for manipulating reformulated OxyContin for abuse during the first few quarters following the product’s launch. However, rather than an increasing level of discussion, we observed a small number of posts related to and mentions of such recipes and a decrease to even smaller numbers over time.

### Strengths and Limitations

Findings from this study should be considered in light of its limitations. Querying Internet posts based on selected keywords is incomplete and does not identify all discussion potentially related to a particular topic. Although the methodology described here has the advantage of providing a systematic and consistent approach over time, it is possible that some discussion associated with OxyContin was missed in this analysis. For example, discussion containing references to the product via terms such as “it,” “that drug,” or “what Joe is using,” in which an individual is making an inference or reference within a conversation may have been missed. This may have introduced selection bias in the sample of posts used. However, it seems unlikely that such bias would result in having completely missed or underestimated significant discussion or topics related to the introduction of reformulated OxyContin and the potential change in OxyContin-related discussion over time.

For the formal content analysis and estimation of endorsement, a high false positive rate was observed for reformulated OxyContin during the postintroduction period (ie, the period following the product’s reformulation), which was primarily attributable to 2 issues. The slang term “OP” (ie, the indicia on the reformulated OxyContin tablet) is also an acronym commonly used on message boards to refer to the “original poster” or the first author to write a post in a thread. Furthermore, because the brand name of the product did not change following reformulation, the search-string queries often yielded posts that, even with human review (which was conducted for this analysis), could not be clearly identified as pertaining specifically to reformulated OxyContin. Although both of these factors contributed to the high degree of false positives, removal of the non–OxyContin-related content as well as ambiguous references to OxyContin that could not be verified as related to the reformulated version of the product ensured that the sample of posts included for analysis in the postintroduction period reflected the target product (ie, the reformulated version) and would, therefore, minimize the effect of misclassification on the results.

It should be noted that references to feasible recipes in this study refer to an author reporting that he/she was able to manipulate reformulated OxyContin and then use it for recreational purposes. Such reports cannot be verified (ie, someone claiming to have tried a recipe may not be telling the truth). However, individuals who participate in the examined forums represent stable communities of drug users and are self-policing so that posted information that is inconsistent with others’ experience tends to be “corrected” by the online community. Additionally, reports of feasible recipe use do not necessarily indicate that the desired effect was achieved as a result of the manipulation. Claims of having abused a manipulated product, whether by an oral or a nonoral route (eg, snorting, injecting), does not mean that the effects were equivalent to, better than, or worse than use of the original product. Although one might expect that the overall poorer sentiment observed for reformulated OxyContin suggests dissatisfaction, the present study did not directly examine satisfaction with results of tampering.

Strengths of this study should be highlighted and include (1) the duration of the study period allowed for a large sample size and examination of trends over time, (2) systematic coding of posts with acceptable interrater reliability, (3) the use of operational definitions of recipe profiles that established a standardized methodology for evaluation of Internet content, (4) the ongoing archiving and storage of Internet posts over time allowed the retrospective evaluation of data and avoided bias introduced by forum moderators deleting older posts for reasons of their own (eg, storage space), and (5) the integration of quantitative (number of Internet posts), content (sentiment of individuals), and qualitative analyses (manipulation recipes) provided a comprehensive approach to understanding the reactions of recreational abusers to the introduction of a tamper-resistant product.

### Conclusions

This study illustrates the value of analyzing Internet discussion on recreational drug use forums to evaluate the impact of introducing a possible tamper-resistant opioid formulation. Introduction of reformulated OxyContin into the marketplace correlated with changes in discussion of abuse-related behavior among recreational abusers as reflected by changes in online conversation levels, reversal of sentiment about the product, and emergence of manipulation-attempt recipes, consistent with findings from other studies showing reductions in abuse and diversion [8-11]. These findings suggest a possible abuse-deterrent effect of the reformulated product relative to the original formulation that was not observed in comparators.

## Abbreviations

ADF: abuse-deterrent formulation
ERo: endorsement ratio
ROA: route of administration
TRF: tamper-resistant formulation
